# Hidden Hearing Loss Impacts the Neural Representation of Speech in Background Noise

**DOI:** 10.1016/j.cub.2020.09.046

**Published:** 2020-12-07

**Authors:** Jessica J.M. Monaghan, Jose A. Garcia-Lazaro, David McAlpine, Roland Schaette

**Affiliations:** 1 National Acoustic Laboratories, Australian Hearing Hub, Macquarie University, Sydney, NSW 2109, Australia; 2 Macquarie University Hearing & Department of Linguistics, Australian Hearing Hub, Macquarie University, Sydney, NSW 2109, Australia; 3 Ear Institute, University College London, 332 Grays Inn Road, London WC1X 8EE, UK

**Keywords:** inferior colliculus, speech in noise, vowel-consonant-vowel, extracellular recordings, classifier, hearing loss

## Abstract

Many individuals with seemingly normal hearing abilities struggle to understand speech in noisy backgrounds. To understand why this might be the case, we investigated the neural representation of speech in the auditory midbrain of gerbils with “hidden hearing loss” through noise exposure that increased hearing thresholds only temporarily. In noise-exposed animals, we observed significantly increased neural responses to speech stimuli, with a more pronounced increase at moderate than at high sound intensities. Noise exposure reduced discriminability of neural responses to speech in background noise at high sound intensities, with impairment most severe for tokens with relatively greater spectral energy in the noise-exposure frequency range (2–4 kHz). At moderate sound intensities, discriminability was surprisingly improved, which was unrelated to spectral content. A model combining damage to high-threshold auditory nerve fibers with increased response gain of central auditory neurons reproduced these effects, demonstrating that a specific combination of peripheral damage and central compensation could explain listening difficulties despite normal hearing thresholds.

## Highlights

• Noise-induced hidden hearing loss caused permanent changes in the auditory brain
• Noise exposure increased neural gain with significantly elevated responses
• Noise exposure impaired discriminability of neural responses to speech in noise
• Model of cochlear synaptopathy and neural gain increase reproduced findings

## Teaser

Monaghan et al. report that noise exposure designed to elicit hidden hearing loss disrupts the neural representation of speech in loud background noise in the auditory midbrain of gerbils. These findings could explain why many people with seemingly normal hearing abilities struggle to understand speech in noisy backgrounds.

## Introduction

Understanding speech is one of the most important roles of the human auditory system. In quiet environments, this task is relatively straightforward, even for individuals whose peripheral auditory system is severely impaired, such as users of cochlear implants [1]. This picture changes dramatically, however, once background noise is introduced; normal-hearing listeners can follow a conversation even when the speech is quieter than the background noise, but hearing-impaired listeners usually require speech to be considerably louder than the background noise in order to comprehend it [2, 3, 4].

Problems understanding speech in noise have long been associated with obvious signs of hearing loss, i.e., elevated hearing thresholds in quiet. However, it is increasingly recognized that individuals whose hearing thresholds are normal can also show unexpected difficulty understanding speech in noise [5, 6, 7]. Recent findings suggest that some of these difficulties might arise from exposure to loud sounds [8], which in animal studies has been shown to cause permanent damage to synaptic contacts between auditory-nerve fibers (ANFs) and the sensory hair cells of the cochlea [9, 10]. This “cochlear synaptopathy” precedes the more commonly considered form of sensorineural deafness associated with damage to, or loss of, the hair cells themselves [11] and leads to a form of hidden hearing loss (HHL)—hidden because it is undetected by conventional tests such as audiometry, which measures the quietest sounds that can be heard. Post-mortem studies of temporal bones have demonstrated direct evidence for age-related synaptopathy in the human cochlea [11]. However, although cochlear synaptopathy is suggested to account for unexplained difficulties processing speech in background noise, to date, no direct evidence of such a deficit has been forthcoming in human listeners [8, 12, 13, 14, 15, 16], for whom noise exposure is largely uncontrolled and only anecdotally reported. Further, investigations of controlled noise exposure on neural responses in experimental animal models have not assessed processing of complex sounds such as speech, being confined largely to relatively simple acoustic signals such as tones (although see [17]).

Here, we demonstrate, in gerbils exposed to a single, controlled noise insult—2 h of octave-band (2–4 kHz) Gaussian white noise presented at 105 dB SPL—evidence of impaired neural coding of speech sounds in background noise in the absence of an increase in neural thresholds to tones. Specifically, 1 month following noise insult, neurons in the auditory midbrain (inferior colliculus [IC]) of exposed gerbils were impaired in their ability to discriminate different vowel-consonant-vowels (VCVs; e.g., AMA, ATA, ASA, etc.) in background noise, compared to animals undergoing a sham exposure. This impairment was evident for VCVs presented at high (75 dB SPL) sound levels and was greatest for VCVs dominated by spectral energy within and above the frequency range of the damaging noise. In contrast, discrimination of VCVs presented at a moderate sound level (60 dB SPL) was, surprisingly, better in exposed than in control animals. A simple phenomenological model of cochlear synaptopathy and enhanced central gain could reproduce this pattern of improved discrimination performance at moderate levels and decreased performance at high sound intensities, linking the effects of peripheral pathology and central plasticity. These results show that noise exposure designed to elicit HHL causes a selective deficit in neural encoding at high sound intensities in the frequency range affected by the noise exposure, which could explain listening difficulties in background noise despite normal hearing thresholds.

## Results

### Response Thresholds of Midbrain Neurons to Acoustic Stimulation Are Not Impaired by Noise Exposure Causing Temporary Shifts in Hearing Thresholds

**Figure 1:**
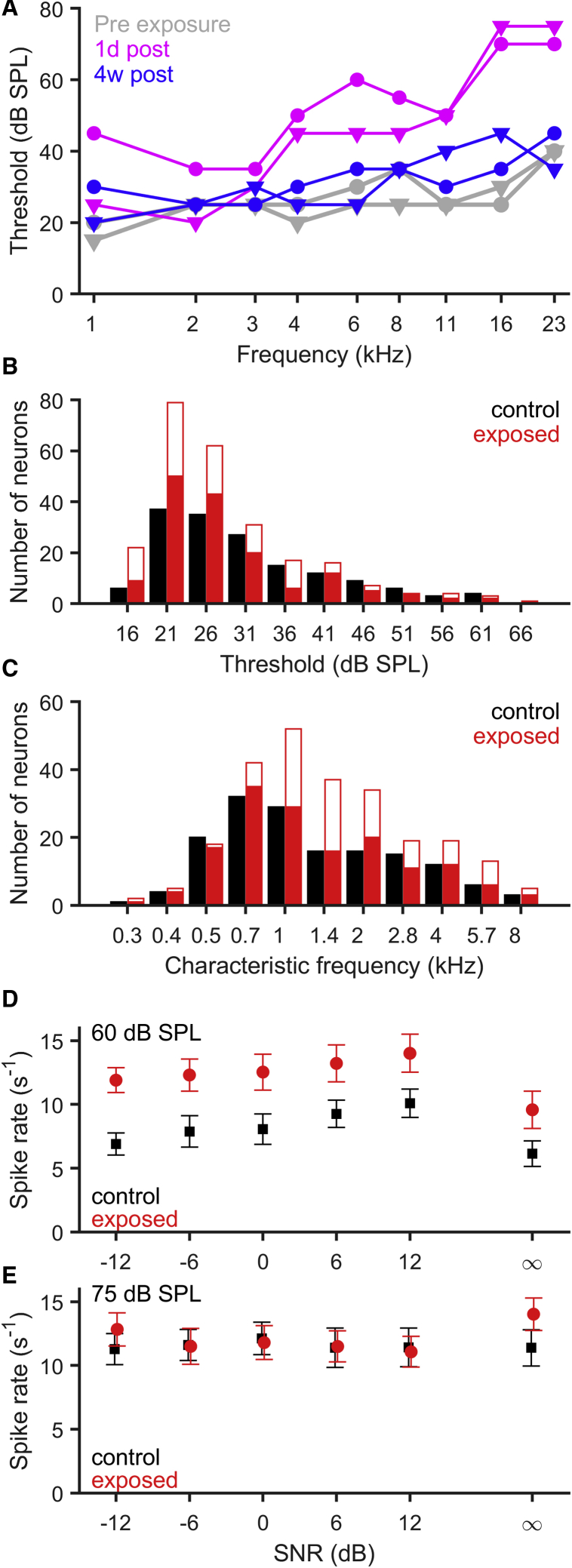
Noise-Induced Hidden Hearing Loss and Inferior Colliculus Recordings (A) ABR thresholds of two example animals before (gray), at 1 day (magenta), and at 30 days (blue) after exposure to octave-band noise (2–4 kHz) at 105 dB SPL. (B and C) Distribution of response thresholds (B) and characteristic frequencies (C) of IC putative single units from noise-exposed (red) and control animals (black). Solid bars illustrate the subset of matched putative single units selected for further analyses. There were no significant differences in thresholds and CFs between recordings from control and exposed animals. (D and E) Average responses of IC putative single units to speech stimuli presented at 60 (D) and 75 dB SPL (E) in different levels of background noise to create SNRs from −12 to 12 dB, and without noise (SNR ∞). Error bars are ± SEM.

We first assessed the impact of noise exposure on basic responses properties of midbrain neurons recorded from anaesthetized gerbils. Neural responses were recorded from the IC to a range of sounds (including pure tones and speech stimuli with and without background noise) from 4 animals exposed to 2 h of 105 dB SPL octave-band (2–4 kHz) noise 4 weeks prior to recording and from 4 control animals subjected to a sham noise exposure. After spike sorting, we identified 291 putative single units (i.e., individual neurons) from the noise-exposed animals and 233 putative single units from control animals. For some units, isolation was lost before all stimulus conditions were presented, and these recordings were excluded from further analysis, leaving a total of 154 putative single units from control animals, and 246 putative single units from exposed animals. To ensure a valid comparison between neural coding of speech sounds in control and exposed animals, neural populations were matched according to their characteristic frequencies (CFs), by selecting 154 putative single units from the exposed group with the closest CFs to each of the putative single units in the control group (where more than one candidate existed, selection was made at random). The distribution of CFs (Figure 1C) was not significantly different between the control and exposed animals for either the original (p = 0.498, Fisher’s exact test) or selected (p = 1.000, Fisher’s exact test) neural populations. Mean thresholds for CF tones in quiet were, surprisingly, slightly but significantly lower in the exposed group (28.1 dB SPL versus 31.0 dB SPL for the CF-based selection; W = 13987, p = 0.00617, Wilcoxon rank-sum test; Figure 1B). Subsequent analyses are based upon these CF-matched populations.

To assess the consequences of noise exposure designed to elicit HHL on speech-in-noise processing, we used a set of 11 vowel-consonant-vowel (VCV) tokens, presented at 60 and 75 dB SPL, in quiet and in the presence of a continuous background of speech-shaped noise presented at 5 different signal-to-noise ratios (SNRs; −12, −6, 0, +6, +12 dB). Analysis of the average firing rates across all VCVs showed that supra-threshold firing rates were significantly higher for VCVs presented at 75 dB SPL compared to 60 dB SPL for control (Friedman test; χ^2^(1) = 11.6, p = 0.000657) but not exposed animals (Friedman test; χ^2^(1) = 0.234, p = 0.628). Moreover, average firing rates were significantly higher in exposed compared to control animals at both sound levels (60 dB SPL: χ^2^(1) = 99.8, p < 1e-16; 75 dB SPL: χ^2^(1) = 15.1, p = 0.000101, Kruskal-Wallis test), but it should be noted that the difference between exposed and control was much more pronounced at 60 (difference in mean spike rate 4.21 sp/s, difference in median spike rate 3.67 sp/s) than at 75 dB SPL (difference in mean spike rate 0.58 sp/s, difference in median spike rate 1.02 sp/s). The combination of normal response thresholds with a pronounced elevation of firing rates at moderate sound levels is consistent with HHL, i.e., noise exposure damaging the integrity of a specific sub-population of ANFs—the high-threshold fibers—in conjunction with an increase in overall neural response gain [17, 18, 19, 20].

### The Neural Representation of Speech in Background Noise Is Impaired in Noise-Exposed Animals with Normal Hearing Thresholds

**Figure 2:**
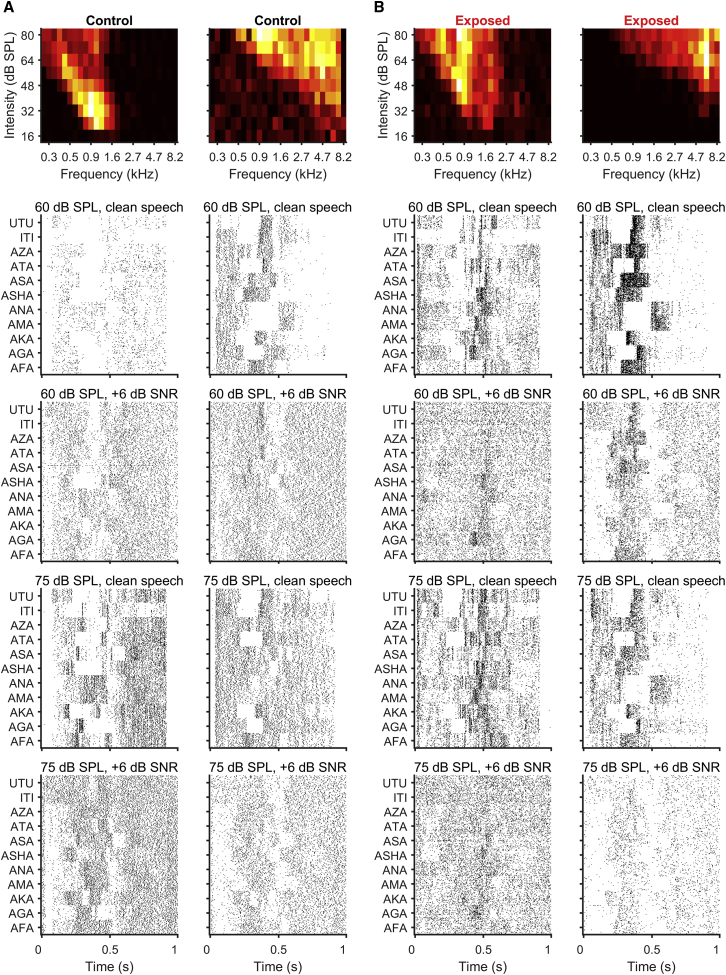
Responses of Individual IC Neurons The frequency versus intensity response area of each example neuron is shown in the top row, and the four rows below show the neuron’s responses to the different VCVs as spike raster plots, for VCVs presented at 60 and 75 dB SPL both without noise and with background noise at +6 dB SNR. Each dot in the raster plots depicts the occurrence of an action potential, and all 32 repetitions are shown for each VCV. (A) Two example units from control animals. (B) Two example units from noise-exposed animals.

To determine whether exposure to loud sounds designed to elicit HHL might underlie problems understanding speech in challenging listening conditions, we assessed the impact of background noise on the neural representation of VCVs in our recordings from both noise- and sham-exposed animals. Dot-raster representations of the responses of representative units to 32 repeats of all 11 VCVs in the absence of masking noise (“clean speech”; Figure 2) and for one of the SNR conditions (+6 dB SNR; Figure 2) indicate that, as background noise was introduced, the neural representation of the VCVs was degraded, evidenced by the reduced structure to the response. This degradation was apparent for VCVs presented at both 60 dB SPL and 75 dB SPL.

**Figure 3:**
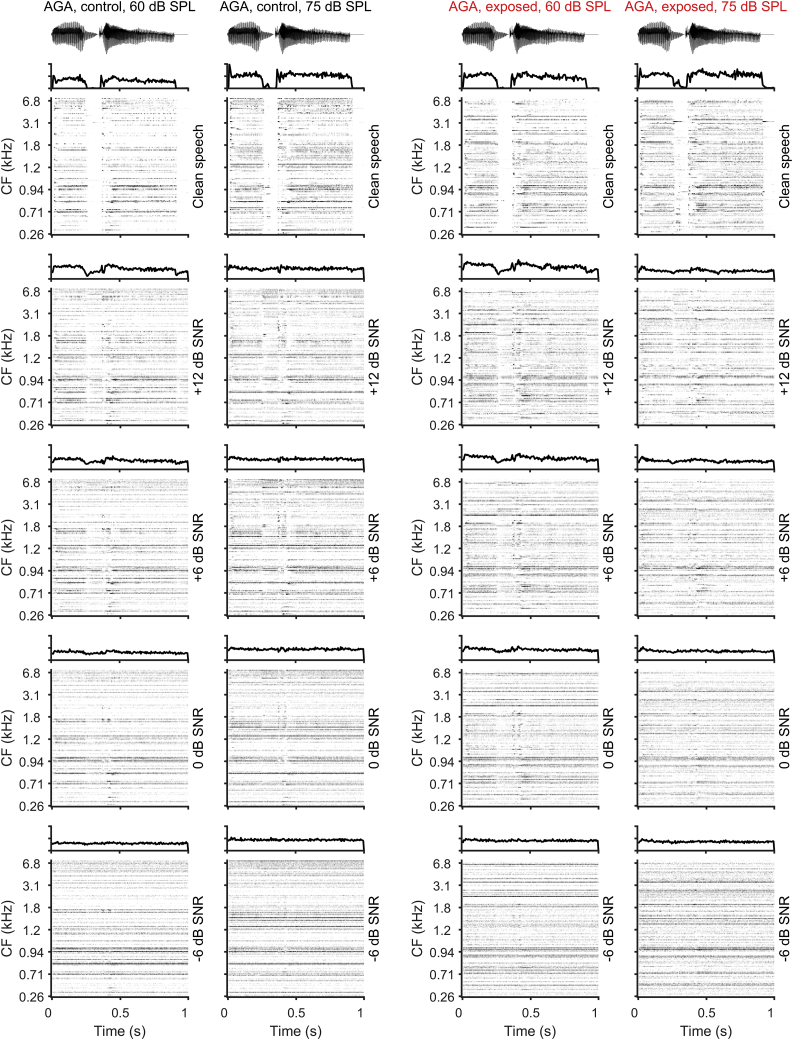
Neurograms—IC Population Responses Average neurograms were constructed by taking the PSTH (1-ms binning) from each of the 154 neurons in each group in response to all repetitions of a specific VCV as a “line” in the neurogram. PSTHs were arranged by characteristic frequency with lower CFs represented at the bottom of each neurogram and increasing CFs at the top. The PSTH across all neurons is shown above each neurogram. This figure shows neurogram for the VCV “AGA,” presented at 60 and 75 dB SPL, for different SNRs (from top to bottom: no noise, +12 dB SNR; +6 dB SNR, 0 dB SNR, −6 dB SNR). The two columns on the left show neurograms from the control dataset for 60 and 75 dB SPL, and the two columns on the right show neurograms from the noise-exposed dataset for the same sound intensities.

To assess the extent to which the neural representation of VCVs is degraded by background noise, we constructed neurograms—representations of the response of the entire neural population visualized in the form of peri-stimulus time histograms (PSTHs) arranged according to each neuron’s CF (i.e., from low to high CFs recorded along the tonotopic axis of the IC). Neurograms for the VCV AGA presented in quiet (clean speech; Figure 3, top row) confirm that the major features of the sound envelopes are clearly represented in average neural responses. At both sound intensities, the neurograms clearly show a diminished response during the brief pause before the onset of the plosive G in AGA. From visual inspection, increasing levels of masking noise (i.e., decreasing SNR, shown for +12, +6, 0, and −6 dB in rows 2–5 of Figure 3), clearly degraded the neural representation of VCVs such that, by −6 dB, the neural response was dominated by the continuous masking noise.

**Figure 4:**
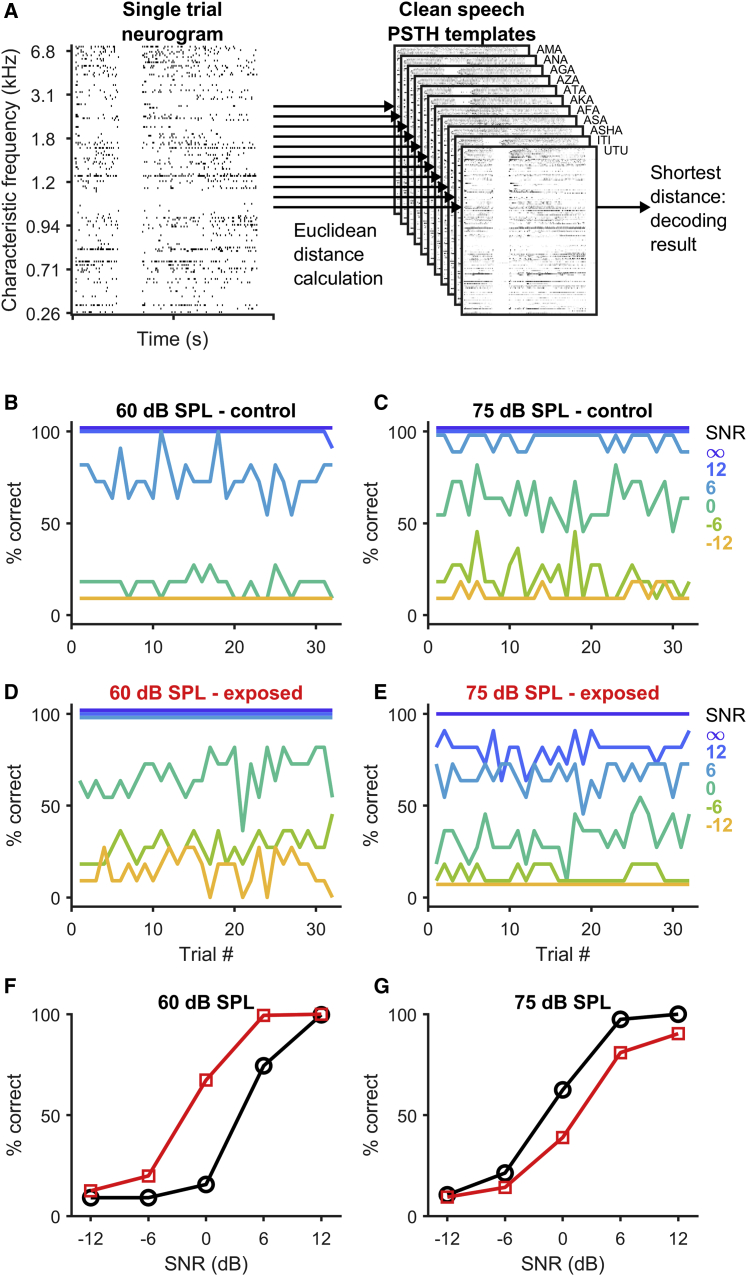
Discrimination of VCVs Based on Neural Responses (A) To discriminate VCVs, single-trial neurograms obtained from each presentation of a VCV in background noise were compared to the average neurogram “templates” obtained for clean speech for all VCVs (STAR Methods, Equation 1). The Euclidean distance was calculated (STAR Methods, Equation 2), and the template with the shortest distance to the single-trial neurogram was taken as the decoding result. (B–E) Average discrimination performance across all VCVs for each of the 32 trials. Results for neurograms from control animals are shown in (B) (60 dB SPL) and (C) (75 dB SPL) and for neurograms from noise-exposed animals in (D) (60 dB SPL) and (E) (75 dB SPL). The different SNR conditions are indicated by the line colors. Please note that some lines have been offset slightly to avoid overlap (e.g., “∞” and “12 dB”). (F and G) Overall discrimination performance versus SNR for VCVs presented at 60 (F) and 75 dB SPL (G), for neural responses from control (black) and noise-exposed animals (red). Confusion matrices are shown in Figure S1, and results for other selections of neurons are shown in Figure S2.

To determine whether prior exposure to noise designed to elicit HHL altered the extent to which neural firing patterns can be used to distinguish different VCVs, we assessed the similarity between pairs of neurograms constructed from the responses to individual VCVs. We hypothesized that neurograms generated in response to different VCVs would be less discriminable from each other in noise-exposed, compared to control, animals. We tested this hypothesis using a PSTH-based classifier with a template-matching procedure based on Euclidean distance metrics (Equations 1 and 2 in STAR Methods). Briefly, the classifier uses a set of templates, one for each VCV, based on the average response of each neuron to the VCV presented in quiet (clean-speech PSTH templates, Figure 4A), and then compares each neurogram obtained from a single trial in background noise to all possible templates. The template closest to the single-trial neurogram in terms of Euclidean distance [21, 22] is then chosen as the classification result (Figure 4A). Responses were assessed over the duration of the entire VCV stimulus, including the response to background noise, from 0 to 1 s in bins of 1-ms duration—a bin size at which responses of gerbil midbrain neurons to human speech sounds appear maximally informative [23]. The outcome of the template-matching process was summed across all VCVs for each combination of SPL and SNR (as well as in quiet) to generate percentage-correct responses for the 32 trials, where each trial consisted of 11 VCVs presented at a single SPL and SNR (Figure 4). In control animals, neural discrimination of VCVs was higher (better) at 75 dB SPL than at 60 dB SPL and systematically fell with decreasing SNR (Figures 4B and 4C). In contrast, discrimination performance in the noise-exposed group was worse at 75 dB SPL than at 60 dB SPL (Figures 4D and 4E). However, surprisingly, discrimination performance at 60 dB SPL was better in the noise-exposed than in the control group at 60 dB SPL (Figure 4F) but worse at 75 dB SPL (Figure 4G). Note that classification performance for clean speech (speech in quiet) was unaffected by sound level or noise exposure (and was always 100%; Figures 4B–4D). Confusion matrices for the VCV classifications based on neural responses are shown in the supplemental material, Figure S1.

To determine which factors and variables significantly impacted the neural representation of speech sounds following exposure to damaging noise, we fitted a logistic regression model for the probability of correct classification with level and exposure as categorical explanatory variables, and SNR and proportion of consonant energy over 2 kHz (hypothesizing that neural discrimination will be most affected for speech sounds with spectral energy at frequencies that are at risk of damage from our noise exposure paradigm) as continuous explanatory variables. All possible interaction terms (up to order 4) were included in the model, and likelihood ratio tests were used to determine the significance of each term in the model.

Significant main effects of exposure (χ^2^(1) = 13.1, p = 0.000295), SNR (χ^2^(1) = 4461.7, p < 1e−16) and high-frequency content (χ^2^(1) = 83.1, p < 1e−16) were observed, as well as significant two-way interactions between exposure and level (χ^2^(1) = 504.8, p < 1e−16), exposure and SNR (χ^2^(1) = 50.9, p = 9.63e−13), exposure and frequency content (χ^2^(1) = 168.3, p < 1e−16), and level and frequency content (χ^2^(1) = 209.1, p < 1e−16). Significant three-way interactions occurred between exposure, level, and frequency content (χ^2^(1) = 9.9, p = 0.00168), between exposure, SNR, and frequency content (χ^2^(1) = 11.1, p = 0.000870), and between level, SNR, and frequency content (χ^2^(1) = 162.2, p < 1e−16). Finally, the four-way interaction between exposure, level, SNR, and frequency content was also significant (χ^2^(1) = 103.8, p < 1e−16). The interaction between exposure and sound level was investigated by fitting a logistic model (with the same explanatory variable as above, except for sound level) separately for each sound level. The effect of exposure was significantly positive at 60 dB SPL (χ^2^(1) = 374.1, p < 1e−16, Bonferroni corrected) and significantly negative at 75 dB SPL (χ^2^(1) = 166.2, p < 1e−16, Bonferroni corrected). Note that, although these results were obtained for a specific selection of 154 putative single units from the exposed group, qualitatively similar results were obtained for other neuron selections, including a selection to obtain matched response thresholds, as well as for completely random selections, or no selection (Figure S2). All of the findings were robust to the specific choice of selected subset, with similar levels of statistical significance seen for all explanatory variables.

Our logistical regression analysis confirms that prior exposure to loud sounds that spares hearing thresholds impairs the neural representation of speech sounds presented at a moderately high intensity (75 dB SPL). This impairment is dependent on the level of background noise; neural discrimination of VCVs presented in quiet was unaffected by exposure but was increasingly impaired as the level of background noise increased compared to neural discrimination in sham-exposed, control animals. In contrast, exposure improved discrimination of speech in noise at moderate (60 dB SPL) sound intensities compared to controls, consistent with the hypothesis that reduced input following damage to high-threshold ANFs leads to elevated gain in the central nervous system [24] and that this might expand the “contrast” in the neural representation of complex stimuli, so long as they fall within the neural dynamic range.

### Noise Exposure Impairs Discrimination of Speech Sounds Most at Risk from Noise Damage

Our inclusion of the frequency content of VCVs as a variable in the regression analysis is based on the hypothesis that neural discrimination will be most impaired for speech sounds whose spectral energy is dominated by frequencies falling within an at-risk range—determined by the frequency content of the noise used to induce damage. For noise-induced hearing loss characterized by damage to sensory hair cells, the greatest damage occurs for frequencies within, and particularly above—where displacement of the basilar membrane is maximal—the frequency range present in the damaging sound [9, 10, 25]. We tested this hypothesis by comparing neural discrimination performance for VCVs as a function of the proportion of their energy lying above the lower edge frequency of the octave-band noise used to induce HHL (i.e., above 2 kHz). Discrimination performance was modeled separately with respect to consonants and vowel energy; 9 of the 11 VCVs were of the form A*x*A, where *x* is the consonant, while 3 (UTU, ATA, ITI) had the same consonant T in combination with a different vowel. For each consonant and vowel, the proportion of energy above 2 kHz relative to the total speech energy was calculated by first extracting the consonant or vowel segment of the original (clean) recording, filtering it to simulate the effect of the ear canal and middle ear in the gerbil [26] and then performing a Fourier transform.

**Figure 5:**
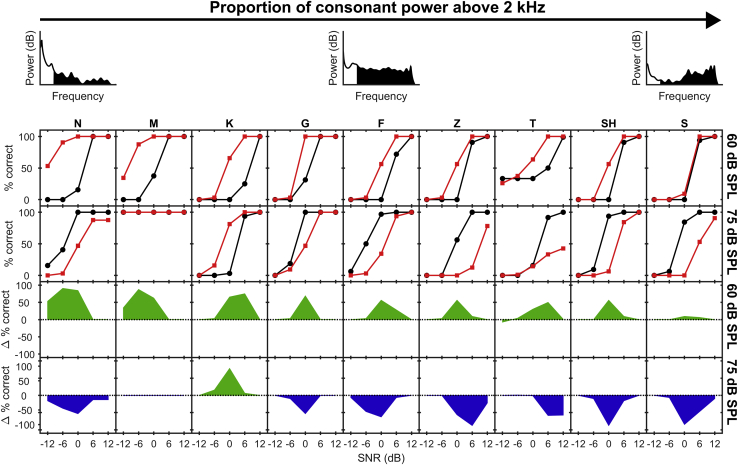
Neural Discrimination Performance by Consonant The two top rows show the neurogram-based discrimination performance for the VCVs AMA, ANA, AGA, AKA, AFA, AZA, ATA, ASHA, and ASA (ordered according to the proportion of consonant power above 2 kHz) for neural responses from noise-exposed (red) and control animals (black). Results for 60 dB SPL are shown in the first row; those for 75 dB SPL are in the second row. The two bottom rows show the performance difference between control and noise exposed, with green signifying better discrimination performance for noise-exposed, and blue better discrimination performance for control. Neural discrimination performance by vowel is shown in Figure S3.

In control animals (Figure 5; black functions in top panels), the consonants S and Sh—which show the highest proportion of energy above 2 kHz—are relatively well discriminated, particularly at 75 dB SPL, as are the consonants M and N—which show the lowest proportion of energy above 2 kHz. In order to determine the effects of noise exposure on discrimination of VCVs with respect to their spectral content, we assessed the interaction between exposure and proportion of high-frequency energy in the fitted logistic regression models for 75 and 60 dB SPL. Discrimination of VCVs presented at 75 dB SPL was most impaired for consonants with the highest proportion of sound energy above 2 kHz. At 75 dB SPL, coefficients in the fitted logistic regression model (assessed for an SNR of 0 dB and for the mean value of the proportion of high-frequency energy) indicated a significant negative association between the estimated effect of noise exposure on the probability of a correct match and the proportion of high-frequency energy (B = −2.48, z = −6.89, p = 5.54e−12). While we observed an overall increase in performance in the noise-exposed group for VCVs presented at 60 dB SPL, (Figure 5), here too there was a significant negative association between the change in probability of a correct match and the proportion of consonant energy above 2 kHz (B = −4.18, z = −10.13, p < 1e−16).

To test the effect of the energy distribution in the vowels (Figure S3), rather than consonants, of VCVs, we employed the same logistic regression approach, but with proportion of vowel energy rather than proportion of consonant energy above 2 kHz as an explanatory variable and the third-order interaction term excluded to avoid perfect separation in the model fitting. The model coefficient describing the effect of noise exposure on the influence of the proportion of high-frequency vowel energy on VCV discrimination was significantly negative at 60 dB SPL (B = −28.4, z = −10.25, p < 1e−16). At 75 dB SPL, the corresponding coefficient was also significantly negative (B = −63.9, z = −10.12, p < 1e−16). In addition, we employed the same logistic regression approach, but with vowel formant-frequency rather than proportion of vowel energy as the explanatory variable. The third-order interaction term and the interaction between formant frequency and SNR were excluded to avoid perfect separation in the model fitting. The model was fitted separately for the second, third, and fourth formants in each vowel, which fall in the frequency range 1,242–4,346 Hz. The model coefficients describing the effect of noise exposure on the influence of formant frequency were significantly negative at 60 dB SPL for the second (B = −0.00369, z = −9.75, p < 1e−16) formant. Similarly, at 75 dB SPL the corresponding coefficients were significantly negative for the second (B = −0.00584, z = −8.65, p < 1e−16) and fourth formants (B = −0.00297, z = −4.60, p = 4.15e−06).

Together, the data suggest that the impact of HHL at high sound levels is frequency-specific; discrimination of speech sounds in background noise—both consonants and vowels—was most impaired for sounds with higher proportions of energy within and above the spectral region defined by the damaging band of noise. This was true even for the moderate sound level, for which the overall effect of exposure was an increase in performance. This suggests that the detrimental effects of noise exposure, which are frequency-specific, might be ameliorated at moderate sound levels by an overall increase in neural gain (see also Figure 1D).

### A Model Incorporating Loss of High-Threshold ANFs and Elevated Gain Accounts for the Effects of Noise Exposure on Neural Discrimination of Speech

**Figure 6:**
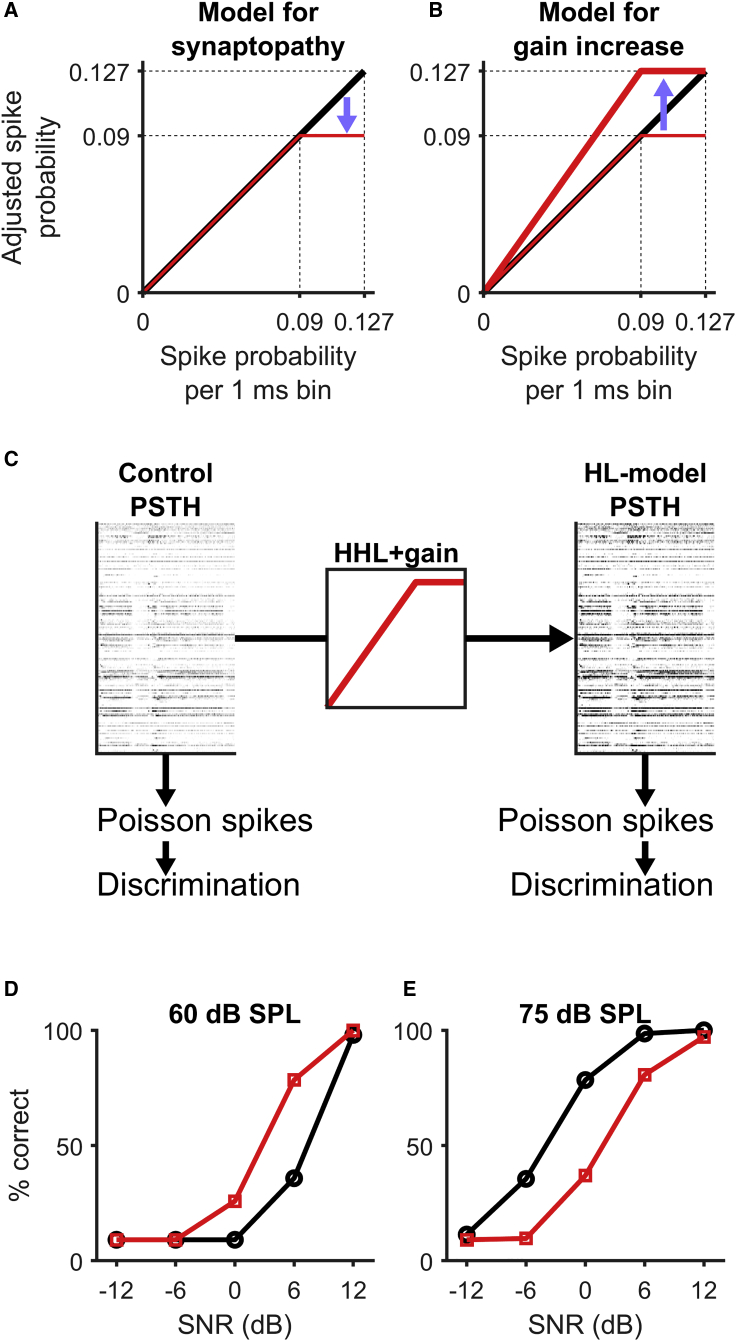
A Phenomenological Model of Synaptopathy and Gain Increase Reproduces the Effects of Noise-Induced HHL on Neural Encoding of Speech (A) The effect of synaptopathy affecting predominantly high-threshold fibers was modeled through a saturation of the spiking probability of IC neurons. (B) Gain increase was modeled through a multiplicative increase of spike probability restoring the maximum spike probability to the normal (non-synaptopathic) value. (C) The HHL+gain function was applied to PSTHs from control animals to generate the corresponding HL-model PSTHs. Poisson spikes were generated from both the control and HL-model PSTHs to generate model neurograms, which were then subjected to the discrimination algorithm to generate model results for the control and hearing loss condition. (D and E) Neurograms from the HL model (red) showed better discriminability than neurograms from the control model (black) at 60 dB SPL (D) but reduced discriminability at 75 dB SPL (E), producing a qualitative match to the results obtained with the experimental neurograms (Figure 4).

We developed a model that captures how the firing probability of auditory midbrain neurons is altered by peripheral synaptopathy and compensatory increase in neural gain in the central auditory system. The simplest form of the model assumes that firing rates saturate at lower sound levels in noise-exposed animals, due to the reduction in input from high-threshold ANFs following noise exposure. To capture this in the model, firing probability is reduced to an asymptote at a value lower than the highest firing probability observed in the control condition (Figure 6A, 1-ms bin size). To optimize the use of the neural dynamic range in the central auditory system, multiplicative gain is then applied such that the saturation point of the function after noise damage and gain increase corresponds to the maximum firing probability of the normal, healthy function (Figure 6B). This HHL-gain function for neural firing probability was then applied to the PSTHs constructed from neural recordings from control animals to generate a model approximation of PSTHs for the noise-exposed condition (HL-model PSTHs).

We then generated model neurograms from both control and HL-model PSTHs (Figure 6C). Briefly, simulated spike patterns were generated by drawing a random number uniformly distributed between 0 and 1 for each bin of the PSTH. If the number was less than the spiking probability for that bin, a spike was deemed to have occurred. This was repeated for 32 trials for each neuron for each condition, and for each VCV. Model spike patterns were subject to the same classification procedure as those generated neurally: model responses for each VCV at each combination of sound level and SNR were compared to simulated templates of responses to clean speech, producing percentage-correct responses for each condition. Consistent with the neural data, the model data indicate that a saturation of input at high sound levels followed by a compensatory increase in gain generates a sound-level dependent effect of HHL; discrimination performance is reduced at 75 dB SPL (Figure 6E) but improved at 60 dB SPL (Figure 6D). The model thus supports the interpretation that deficits in speech-in-noise discrimination by midbrain neurons at high sound levels (75 dB SPL) can be explained by a relative loss of responses of high-threshold ANFs, and that the surprising observation of an improvement in speech-discrimination performance at moderate sound levels (60 dB SPL) can be a result of elevated neural gain in the central auditory system following noise exposure.

## Discussion

We have demonstrated evidence of impaired coding of speech in background noise in animals exposed to noise designed to induce so-called hidden hearing loss (HHL), i.e., noise exposure causing only a temporary hearing threshold shift (TTS) with full recovery of hearing thresholds over time, but permanent damage to structures of the inner ear. Our data are consistent with the interpretation that damage to ANFs following exposure to loud sounds impairs the neural representation of speech in background noise in the central auditory nervous system, even when hearing thresholds are spared. Consistent with the reported pattern of damage to ANFs [9, 10], impaired speech-in-noise performance is evident only at relatively high sound levels, and speech sounds with energy within and above the band of damaging noise are most affected. Conversely, at moderate sound levels, we observed a paradoxical improvement in the neural representation of speech sounds following exposure, likely the result of elevated neural gain in the central pathways in response to reduced input [24, 27, 28]. These findings corroborate the hypothesis that the effects of noise-induced HHL may be sound-level specific [18] and therefore difficult to detect in humans when standard speech-in-noise tests are administered only at a “comfortable” sound level.

Different structures of the inner ear may be affected in HHL: cochlear synaptopathy [10, 29], cochlear neuropathy [10, 17, 30], and even scattered loss of inner hair cells [31] have been observed in ears of animals with normal hearing thresholds after noise exposure or administration of ototoxic drugs. Compared to control animals subjected to a sham exposure, neurons in the auditory midbrain (IC) of noise-exposed gerbils showed significantly poorer neural discrimination of speech sounds (VCVs) in loud background noise. The effect of exposure differed markedly between the two stimulus levels, impairing discrimination at 75 dB SPL but improving it at 60 dB SPL. The impairment in discrimination performance at 75 dB SPL was greatest for VCVs with consonants whose spectral content was dominated by frequencies within or above the 2–4 kHz frequency band of damaging noise, i.e., in frequency regions where noise damage would be expected. Discrimination of VCVs in quiet was not impacted by noise exposure. This pattern is consistent with electrophysiological evidence that high-threshold ANFs are more susceptible to noise damage than medium- and low-threshold ANFs [9], although the relative sensitivity of the different fiber types to noise exposure is currently a matter of some debate. Noise-induced synaptopathy will reduce the number of ANFs providing input to the central auditory system, thus lowering the effective signal-to-noise ratio of the neural signal and increasing the variance of the input received by the IC, with potential detrimental effects on the fidelity of neural representations. Noise-induced damage to high-threshold ANFs could also account for our finding that average response thresholds of IC neurons to pure tones were even slightly lower in the exposed compared to the control group: IC neurons relying exclusively on high-threshold ANFs as input (via intermediate relays) might have been silenced after noise exposure, and sampling from the remaining unaffected neurons would then yield a lower threshold.

In contrast to performance at the higher sound level (75 dB SPL), neural discrimination performance for VCVs presented at 60 dB SPL was significantly better for neurons recorded from exposed, compared to control, animals. Taken with evidence that noise exposure that elicits a temporary elevation of hearing thresholds generated increased firing rates at moderate sound levels (Figure 1D), this improvement is consistent with the hypothesis that reduced neural output of the noise-damaged cochlea and auditory nerve leads to elevated gain in the central auditory nervous system [24, 27, 28] and that this elevated gain can improve listening performance at moderate sound levels. Notably, unlike the deficits observed at the higher sound level, improved discrimination performance was not restricted to speech sounds with relatively higher spectral energy but was evident across the frequency gradient. This suggests that one response to reduced neural input following noise damage is a compensatory, potentially homeostatic, increase in neural gain to compensate for this loss of input. A similar increase in gain across a wide range of frequencies above and below the noise exposure band, i.e., not limited to frequency regions affected by cochlear damage, has, for example, been reported for the auditory cortex after noise exposure [32]. The IC receives multiple ascending and descending convergent inputs—including from both ears—making it an ideal site in which to assess system-level changes, such as overall neural gain, following altered sensory input. However, this convergence militates against determining more subtle factors, including in peripheral hearing function, that might also contribute to altered speech-in-noise processing. Sub-lethal noise exposure might broaden cochlear filters, for example, without elevating hearing thresholds (or, at least, beyond the range considered audiometrically normal). Although likely subtle—and difficult to assess using current diagnostic tools—broadening of cochlear filters might be expected to contribute to problems listening in noise, even in the absence of elevated hearing thresholds.

The notion that reduced auditory-nerve output in humans may lead to increased neural gain (and hearing pathologies) has been demonstrated in listeners with tinnitus but otherwise normal hearing thresholds. Compared to matched controls without tinnitus, listeners with tinnitus showed reduced magnitude auditory brainstem response (ABR) wave I—generated by the auditory nerve—at high sound intensities [24, 33, 34], consistent with the cochlear synaptopathy. Neural activity in the midbrain (wave V of the ABR), however, was of normal [24, 34] or even increased [33] magnitude, suggesting a compensatory increase in neural gain had offset reduced neural input, with generation of tinnitus potentially a side effect of this central compensation. Direct support of this gain hypothesis comes from a recent animal study demonstrating that severe selective ablation of ANFs with the drug ouabain elicits an increase in neural gain in the auditory midbrain and cortex [17], but that this increase in gain is not sufficient to restore neural coding of complex signals such as speech.

To assess the discriminability of neural responses to speech sounds, we have used a nearest-neighbor classifier based on a Euclidean distance metric [21]. For responses recorded from the auditory cortex of normal-hearing rats, results obtained with this classifier showed a high correlation to behavioral performance for speech sounds presented in quiet [22]. A modified version of the classifier also showed a good correspondence between neural and behavioral discrimination performance in background noise [35]. Interestingly, a recent study reported an increase in the average Euclidean distance between cortical activity patterns in response to consonant sounds presented at 60 dB SPL for rats with moderate noise-induced hearing loss [36], similar to our own data, although these authors did not report whether the increase in Euclidean distance changed discriminability. Finally, it has been demonstrated that behavioral and cortical neural discrimination performance in normal-hearing rats is close to that of human performance [37, 38], supporting the notion that the changes in neural discrimination performance we observed in the gerbil IC following noise exposure may be predictive of changes in listening performance in humans with HHL.

Impaired neural coding of complex sounds due to HHL could explain problems many otherwise normal-hearing individuals experience when trying to understand speech in disadvantageous listening environments such as noisy restaurants, railway stations, or busy streets. Despite some promising recent results linking HHL to decreases in listening performance [8, 39, 40, 41], a considerable degree of uncertainty remains concerning the extent to which HHL might affect listening performance in humans [13, 42, 43, 44], and diagnostic measures of synaptopathy or other manifestations of HHL have yet to be established [12]. Since direct anatomical evidence of cochlear synaptopathy and neuropathy can only be obtained *post-mortem* [11], clinical assessment of the degree of HHL is likely only to be based on correlations between listening performance and (yet to be determined) electrophysiological measures of neural activity. This is exacerbated by the fact that data pertaining to life-time noise exposure are largely anecdotal and difficult to quantify. Investigations in animal models are therefore required to progress new diagnostic approaches in humans.

Our results indicate that speech-in-noise tests could be made more sensitive to the potential effects of HHL by testing at both moderate and loud sound intensities. We predict that subjects with HHL show an abnormally large reduction in performance as presentation level is increased. In contrast, when testing is performed simply at a “comfortable” sound level, as is commonly the case, subjects with HHL might perform even better than expected. It remains to be determined whether frequency-specific effects of noise exposure seen in our animal model might also be exploited to develop a more specific listening test; HHL might be distributed more evenly along the length of the cochlea in humans [11, 45], as any damage in human cochleae is more likely the consequence of exposure to a variety of loud sounds over a lifetime, compared to a single insult with a stimulus of limited frequency range employed experimentally. Finally, our data also suggest a therapeutic intervention for listeners who struggle following a conversation in challenging listening conditions despite normal hearing thresholds: a device that attenuates (rather than amplifies, as in a standard hearing aid) high-level sounds might bring them into the range over which neural coding might not only be preserved, but potentially enhanced, after HHL, and thus improve listening performance in loud background noise.

## STAR★Methods

### Key Resources Table

**Table:** 

REAGENT or RESOURCE		SOURCE	IDENTIFIER
**Deposited Data**			

Raw data	This study		https://doi.org/10.17632/s9856wfgdd.1

**Experimental Models: Organisms/Strains**			

Gerbils (Meriones unguiculatus)	Charles River		N/A

**Software and Algorithms**			

R	https://www.r-project.org/		N/A
MATLAB	Mathworks		N/A
KlustaKwik	http://klustakwik.sourceforge.net		N/A
Klusters	[46]		N/A
Neurogram discrimination algorithm	This study		https://doi.org/10.17632/s9856wfgdd.1

**Other**			

32 channel tetrode arrays	Neuronexus Technologies, Ann Arbor, MI, USA		N/A

### Resource Availability

#### Lead Contact

Request for further information and resources should be directed to and will be fulfilled by the Lead Contact, Roland Schaette (r.schaette@ucl.ac.uk).

#### Materials Availability

This study did not generate new unique reagents.

#### Data and Code Availability

The datasets and code generated during this study are available under https://doi.org/10.17632/s9856wfgdd.1

### Experimental Model and Subject Details

Subjects for the animal experiments were adult, male Mongolian gerbils (*Meriones unguiculatus*) with typical weights between 70-90 g, and ages ranging between 3-6 months, which were randomly assigned to experimental groups. The experimental protocols described in this section were approved by the United Kingdom Home Office Inspectorate under project license 30/2481, in conformity with the 1986 Animals Scientific Procedures Act.

### Method Details

#### Protocol Timeline

Briefly, on day 1, auditory brainstem responses (ABRs) were recorded to estimate the hearing thresholds of animals contributing as subjects. On day 2, animals were exposed to high-intensity (105 dB SPL) noise band-pass filtered between 2 and 4 kHz, for a period of two hours. Auditory brainstem responses were recorded on day 3 to confirm that noise exposure elicited an elevation of hearing thresholds. On day 30, another set of ABR recordings was carried out, followed by recording of extracellular responses of neurons in the inferior colliculus (IC) using multi-electrode arrays.

#### Auditory Brainstem Response Measurements

For ABR measurements, animals were anesthetized via intraperitoneal injection of a mix that consisted of fentanyl/medetomidine/midazolam (ratio 1.6/0.4/10 respectively). Additional doses were administered throughout the experiment as required by assessing the level of anesthesia using the pedal withdrawal reflex. Subdermal needles (Rochester Medical) were used as electrodes and were inserted at the vertex and one each behind the ipsilateral and contralateral pinnae respectively. Throughout the recordings the body temperature was maintained constant between 37–38°C using a homeothermic blanket (Harvard Apparatus, Cambridge, UK). Acoustic stimuli consisted of either tone pips (5 ms total duration and 1.5 ms rise/fall time) with frequencies set to 1024, 2048, 2896, 4096, 5793, 8192, 11585, 16384 and 23170 kHz presented at varying intensities from 0-80 dB SPL in 5 dB steps, or clicks (50 μs duration, 0-80 dB SPL in 5 dB steps) delivered at a rate of 20/s. Stimuli were generated, attenuated and amplified using TDT system 3 (Tucker Davis Technologies, Alachua, FL, USA) and presented in a free-field manner via a TDT CF1 speaker positioned at a 45° angle relative to the animal’s axis, and at a distance of approximately 18 cm. During the recordings, the ear contralateral to the speaker was temporarily blocked using a foam earplug. Electrode signals were low-pass filtered (7.5 kHz cut-off frequency, 12 dB per octave) and recorded at a sampling rate of 24 kHz using TDT system 3 hardware. For analysis purposes, the data were filtered using a bandpass filter (100-3000 Hz, 5th-order Butterworth filter). Once the ABR recordings were complete, an intraperitoneal injection consisting of a mix of atipam/flumazenil/naloxone (ratio 1/50/15.6 respectively) was administered to the animal, which was subsequently placed in a temperature-controlled recovery chamber for approximately one hour before returning it to the housing facility.

Stimuli were calibrated using a 1/4-inch microphone (G.R.A.S., Skovlytoften, Denmark) placed at the location where the animal’s ear would be during the recordings. A filter was applied to adjust the frequency response of the speaker such that it was flat (±3 dB) between 1 and 24 kHz. ABR thresholds were determined visually by estimating the lowest sound level at which deflections in the ABR waveform were judged to be greater than the background variability in the waveforms. Measurements of wave amplitudes were performed using custom written MATLAB software (Natick, MA, USA). Briefly, a time window containing the wave of interest was defined and the maxima and minima of the traces were estimated within this window. ABR wave amplitudes were measured from the peak to the following trough.

#### Noise Exposure

Anesthesia in gerbils was induced by intraperitoneal injection of fentanyl/medetomidine/midazolam (using the same ratio as above). Additional top up doses were delivered as required by assessing the pedal withdrawal reflex. Body temperature was maintained constant at 37-38°C and the respiratory rate was checked every 30 minutes. Animals were placed in a custom-made sound-proof booth directly under the center of a speaker (Monacor Stage Line PA Horn Tweeter MHD-220N/RD, Bremen, Germany) positioned 45 cm above. The speaker was calibrated prior to each use to ensure that the frequency response was flat (±2 dB) over the 2-4 kHz range. Animals were exposed to octave-band noise (2-4 kHz) at 105 dB SPL over a period of two hours. Stimuli were generated, attenuated and amplified using TDT system 3 hardware. After noise exposure, animals were administered an intraperitoneal injection of a mix which consisted of atipam/flumazenil/naloxone (using the same ratios as described above) and were allowed to recover for approximately 1 hour in a temperature-controlled chamber. For sham exposures, the speaker was left unplugged.

#### Single-Neuron Recordings

Experiments were conducted in a sound-insulated chamber (Industrial Acoustics, Winchester, UK). Anesthesia in gerbils was induced by intra-peritoneal injection of 1 mL per 100 g body weight of ketamine (100mg/ml), xylazine (2% w/v), and saline in a ratio of 5:1:19 as described in [47]. The same solution was infused continuously during recording at a rate of approximately 2.1 μl/min. Body temperature was maintained at 38.7°C by a homeo-thermic blanket controlled via feedback from a rectal probe. The skull was exposed by incision of the scalp and a metallic pin was cemented to it. The pin was subsequently coupled to a stainless-steel head-holder in a stereotaxic frame. A craniotomy was performed on the right side of the skull extending 3.5 mm from the mid-line and centered along the lambdoid suture. To improve the separation of single units, we used 32-channel silicon array electrodes (Neuronexus Technologies, Ann Arbor, MI, USA) arranged in tetrodes with 2 tetrodes in each of the four shanks of the probe.

To prevent drift over time caused by the displacement of brain tissue along the shanks, electrodes were initially inserted into the overlying cortex after removal of the dura, and guided through at a speed of 1 μm/sec, and then slowed to 0.3 μm/sec upon contact with the IC in order to ensure tissue displacement was minimal, until all recording sites were located inside the central nucleus of the IC (as determined by tonotopically ordered tuning curves recorded on the electrode arrays). After the responses to all the stimuli in our set were recorded, the electrode was advanced ca. 300 μm to a deeper location. Typically, at least three different electrode penetrations were made along the rostral-caudal axis (at 150-μm intervals), with the data collected at two different depths in each penetration. Human speech is dominated by sound-frequencies typically lower than 8 kHz, and we therefore aimed to record responses from neurons with comparable low CFs, which are located more superficially in the ICc. Oxygen-enriched air was delivered to the vicinity of the snout and ECG and body core temperature were monitored throughout the duration of the experiment.

For spike sorting, we followed established procedures described in detail in [48]. Briefly, the method consists of (1) bandpass filtering each channel between 500 and 5000 Hz; (2) whitening each tetrode, i.e., projecting the signals from the four channels into a space in which they are uncorrelated; (3) identifying potential spikes as snippets with energy [49] that exceeded a threshold (within a minimum of 0.7 ms between potential spikes); (4) projecting each of the snippets into the space defined by the first three principal components for each channel; (5) identifying clusters of snippets within this space using KlustaKwik (http://klustakwik.sourceforge.net) and Klusters [46]; and finally, quantifying the likelihood that each cluster represented a single unit using an isolation distance criterion [50]. The isolation distance assumes that each cluster forms a multi-dimensional Gaussian cloud in feature space and measures, in terms of the SD of the original cluster, the increase in the size of the cluster required to double the number of snippets within it. The number of snippets in the “noise” cluster (non-isolated multiunit activity) for each tetrode was always at least as large as the number of spikes in any single-unit cluster [48]. Only single-unit clusters with an isolation distance > 20 were considered for further analysis. On average, each tetrode yielded between 1-2 single-units which amounts to approximately 8-16 single-units per electrode penetration. After this stage, units lost during the presentation of our stimulus set, or units in which the CF could not be reliably determined, were further excluded from the analysis.

#### Stimuli

To characterize the basic response properties of single neurons in the IC, we recorded frequency response areas (FRAs) using 50-ms pure-tone pips of varying frequencies (250 Hz to 8.192 kHz) and intensities (20 to 80 dB SPL) presented once every 150 ms.

To investigate the neural representation of speech-in-noise, we assessed neural responses evoked by repeated (32) presentations of 11 different vowel-consonant-vowel (VCV) tokens from an adult female speaker. A window function with a 5-ms ramp was used to limit the VCVs to 0.9 s duration, and VCVs were presented at 1 s intervals at varying signal-to-noise ratios (−12 to 12 dB, in steps of 6 dB). Nine of the VCVs took the form ‘A*x*A’; i.e., the vowel was A, and the consonants were M, N, G, Z, T, K, F, S, Sh, (e. g., ‘AMA’, ‘ANA’, etc.). Responses to two further vowels of the form ‘*x*T*x*’ were assessed (‘ITI’ and ‘UTU’), providing a further comparison of three different VCV combinations (‘ATA’, ‘ITI’, and ‘UTU’) with the same consonant. VCVs were presented at a sound level of 60 or 75 dB SPL, and in various levels of speech-shaped background noise to create SNRs of −12, −6, 0, +6 and +12 dB.

#### Classifying Neural Responses to VCVs

For some neurons, it was not possible to record all stimulus conditions, and these neurons were excluded from further analysis. Additionally, some units had extremely sparse responses to tones, and thus a CF could not be determined reliably. These units were also excluded, leaving a total of 154 neurons from control animals, and 246 neurons from exposed animals. In order to enable a valid comparison between neural coding of speech sounds in control and exposed animals, neural populations were matched according to their characteristic frequencies, and thus 154 neurons were selected from the noise-exposed group.

The PSTH classifier we used for this analysis is described in detail in [21]. Briefly, single-trial responses were grouped in sets of *S* possible stimuli (n = 11). Each stimulus in the set was presented *T* (n = 32) times during the experiment while the activity of *N* single neurons was recorded. For every neuron, a 1 s time window following the stimulus onset was divided into B 1-ms bins containing spike counts with the desired temporal resolution. The dataset thus consists of a matrix *T* x *S* rows and *B* x *N* columns (see Figure 2 in [21]). $v_{i,j}$ denotes the spike counts in the *i*th row and the *j*th column of the matrix, where *i* goes from 1 to *ST* (total number of stimuli x trials per stimulus) and *j* goes from 1 to *NB* (number of neurons x number of bins). The template for each stimulus *s* is defined by ${\bar{v}}^{s}=\;\left[{\bar{v}}^{s},\ldots ,\;{\bar{v}}_{NB}^{s}\right]$, and the *j*th element is calculated as

(Equation 1) $${\bar{v}}_{j}^{s}=\;\frac{1}{T}\sum_{i\;\in \;s}v_{ij}^{s},$$

where *T* represents the total number of trials. The templates were generated from responses to the clean VCV at each sound level and separately for each exposure condition. An equal number of neurons (154) were used in each level and exposure condition. For each single trial, $v_{i}=\;\left[v_{i,1},\ldots ,\;v_{i,NB}\right]$, the Euclidean distance between the single trial itself and each stimulus template ${\bar{v}}^{s}$ is defined as

(Equation 2) $$d_{s}^{i}=\sqrt{\sum_{j=1}^{NB}{\left(v_{i,j}-\;{\bar{v}}_{j}^{s}\right)}^{2}}.$$

Single trials were classified to the template with the smallest distance. To avoid artifacts the trial being tested was not used to generate the template when the clean conditions were assessed.

#### Phenomenological Model of Cochlear Synaptopathy and Enhanced Central Gain

To test the hypothesis that synaptopathy and a central gain mechanism could account for the pattern of results seen in the neural data from the exposed animals, a simple model was used to transform the data from the unexposed animals (Figure 6). The effect of synaptopathy was modeled with a broken-stick such that the firing probability was saturated above the knee-point, truncating the top of the dynamic range. It was hypothesized that the function of the central gain would be to normalize the firing rate such that the maximum firing rate after the synaptopathy model plus the compensatory gain was applied would be equal to the maximum firing rate of the neurons before exposure. The maximum firing probability was taken as the mean of the maximum firing probability—0.127—across all neurons in the 75 dB SPL (non-exposed) condition. The knee point of the function was determined from the observed gain between firing rates across all SNRs (including quiet) for control and exposed conditions at 60 dB SPL, equivalent to 1.40. The reciprocal of the gain (0.716) multiplied by the maximum firing probability generates the saturation point of the function (0.09) (Figures 6A and 6B). Application of this “HHL-gain”-function to the PSTHs of responses from control animals yielded “HHL-model” PSTHs (Figure 6C). Both control and HHL-model PSTHs, for all VCVs and all SNR conditions, were then used as input to a Poisson process with variable rate to generate artificial spike trains, which were then assembled into model neurograms and analyzed for their discriminability using the PSTH-based classifier (Figure 6C).

### Quantification and Statistical Analysis

#### Data Analysis and Statistical Testing

Characteristic frequencies and response thresholds of IC neurons were determined from the FRAs by a trained observer who was blinded to the experimental group. The VCV classification analysis was performed in MATLAB using a custom script. All statistical analysis was performed in R (version 3.6.1). The performance of the classifier in terms of whether each presentation of a VCV was correctly or incorrectly classified was modeled using binomial logistic regression (glm function). The fitted model took level and exposure as categorical variables and SNR and proportion of consonant or vowel energy over 2 kHz as continuous variables. All interaction terms were included in the model and the likelihood ratio test was used to determine the significance of each term in the model using the ‘Anova’ function from the car package (version 3.0-6). The significance of the coefficients in the model was evaluated using the Wald test. Analysis of the similarity of the distribution of the CFs for the populations of neurons was performed using Fisher’s exact test (‘fisher.test’) and analysis of the similarity of the threshold distributions was performed using the Wilcoxon rank sum test with continuity correction (‘wilcox.test’). Spike rates were tested for normality using the Shapiro-Wilks test (‘shapiro.test’ in the ‘stats’ package, version 3.6.2), and since the assumption of normality was violated, non-parameteric statistics were used to test differences between the spike-rate distributions with level (using the Kruskal-Wallis test, kruskal.test) and exposure status (using the Friedman test, friedman.test). All error bars in figures are ± SEM.

## Supplemental Information

Document S1. Figures S1–S3

Document S2. Article plus Supplemental Information
