# Supplementary material for: Hidden Hearing Loss Impacts the Neural Representation of Speech in Background Noise
Source: Curr Biol. 2020 Dec 7;30(23):4710–4721.e4. doi: 10.1016/j.cub.2020.09.046 (PMC7728162; doi:10.1016/j.cub.2020.09.046)
Supplement: Document S1. Figures S1–S3 [file mmc1.pdf]

Current Biology, Volume *30*

Supplemental Information

Hidden Hearing Loss Impacts the Neural Representation of Speech in Background Noise

Jessica J.M. Monaghan, Jose A. Garcia-Lazaro, David McAlpine, and Roland Schaette

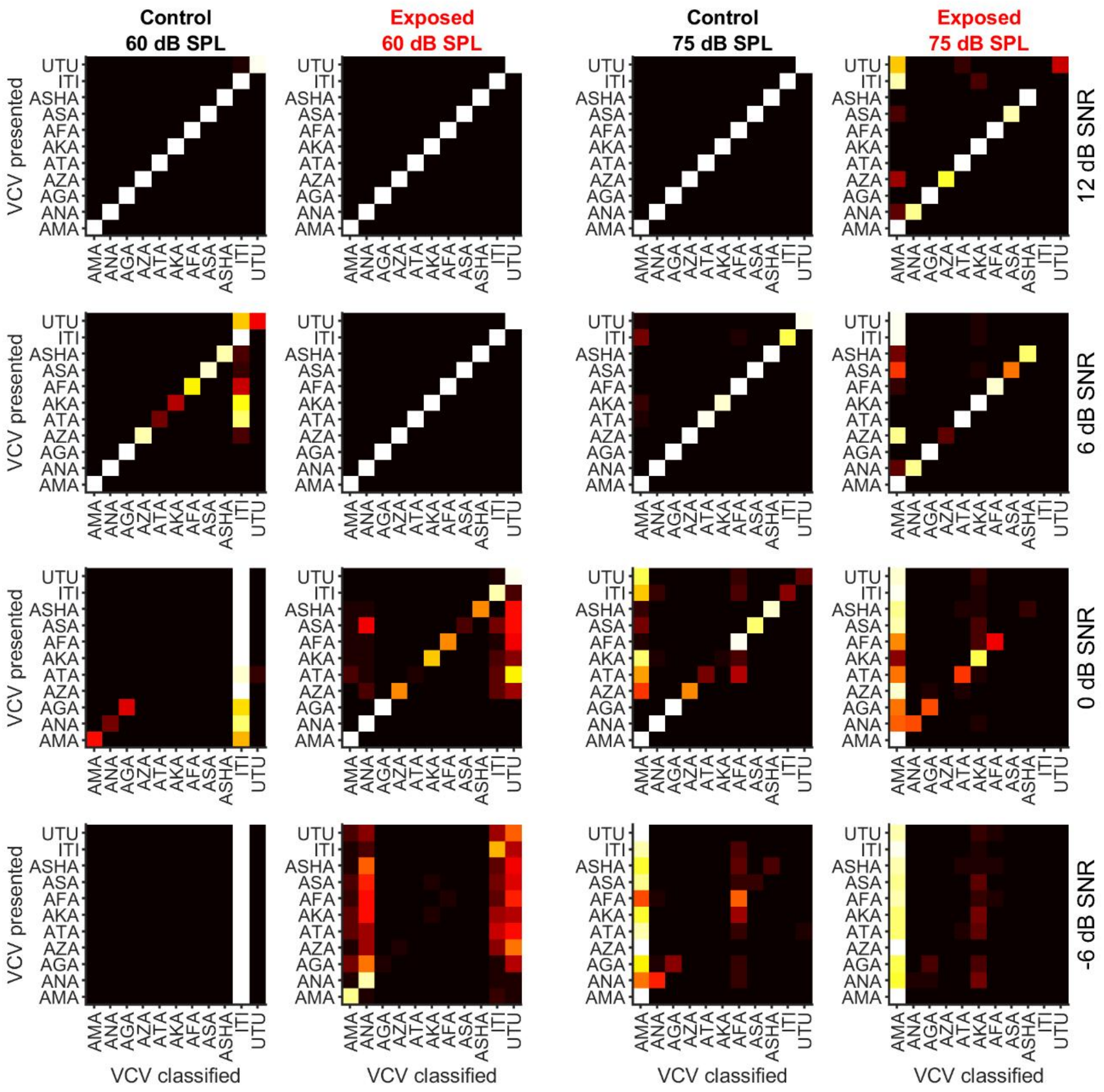

Figure S1: Neural discrimination performance: confusion matrices. Related to Figure 4. In the matrices, the colour of each square (referenced to the heat map) indicates the number of times, out of 32 possible trials (see Figure 4), that the response obtained to the presentation of one VCV (y-axis) is classified to every other VCV in the set (x-axis). Perfect discrimination is thus indicated by white squares on the diagonal. The two columns on the left show results for 60 dB SPL speech presentation level for neural responses from control and noise-exposed animals, respectively, and the two columns on the right show results for 75 dB SPL speech presentation, again for control and noise-exposed data. Four different SNR conditions (+12, +6, 0, and -6 dB SNR) are depicted in rows from top to bottom.

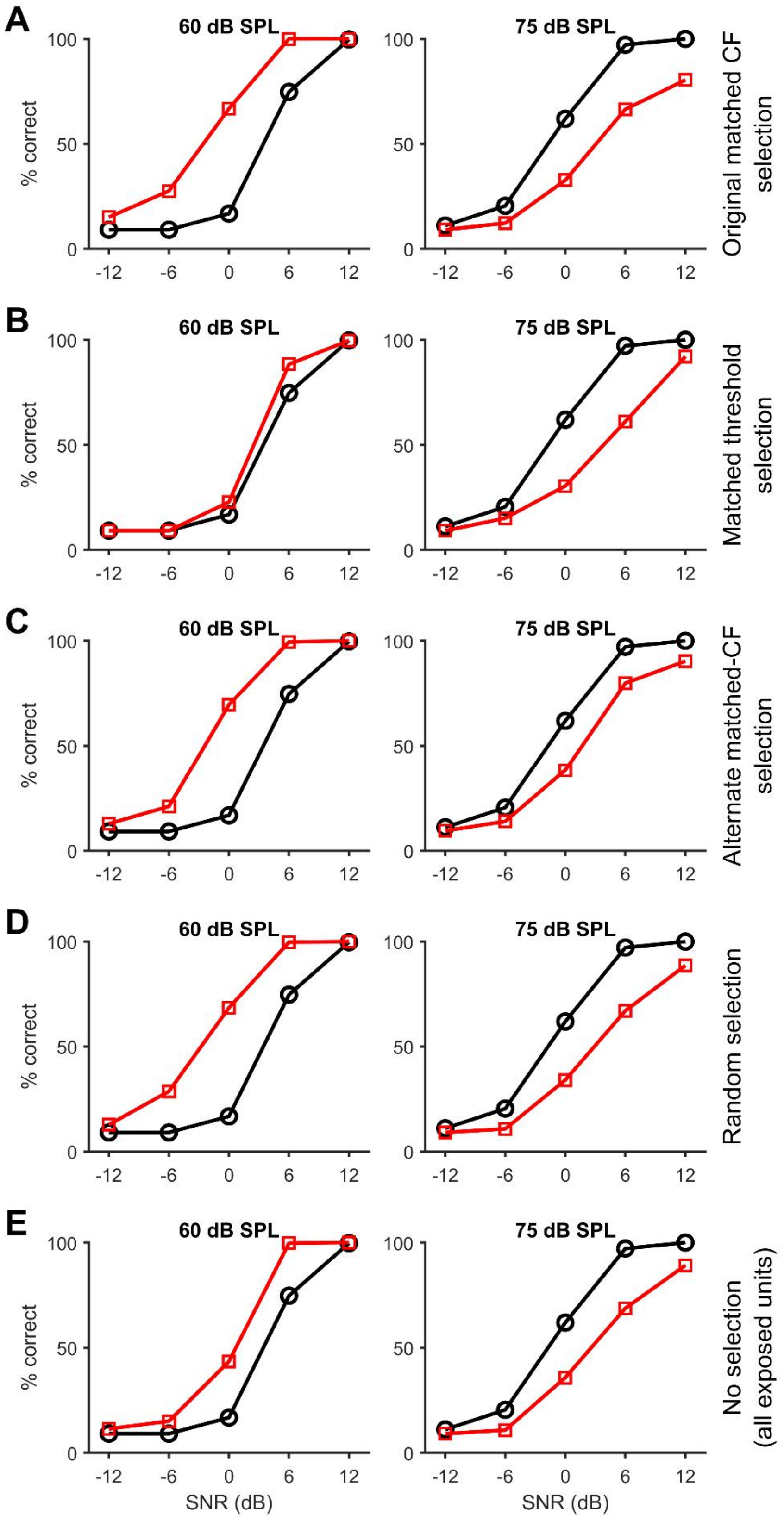

Figure S2: Neural discrimination performance for different neuron selections. Related to Figure 4. a) Results for the neuron selection for matched characteristic frequencies used in the main paper. b) Results for a selection of 154 neurons from the exposed group to match neural response thresholds

between control and exposed group. c) Results for an alternative selection of 154 neurons to achieve matched CFs between control and exposed group. d) Results for a completely random selections of 154 neurons from the exposed group. e) Results for neural discrimination based on all 246 neurons from the exposed group. While overall discrimination performance for discrimination based on the neural responses from the exposed group differed slightly across the different selections, the general pattern of improved performance at 60 dB SPL and decreased performance at 75 dB SPL was seen for every selection.

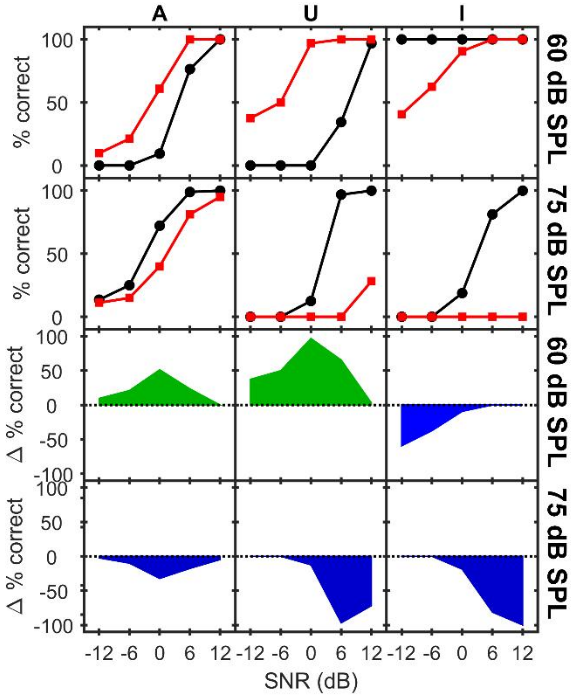

Figure S3: Neural discrimination performance by vowel. Related to Figure 5.

The two top rows show the neurogram-based discrimination performance for the three VCVs 'ATA' (left), 'UTU' (middle), and 'ITI' (right) presented at 60 (row 1) and 75 dB SPL (row 2), for neural responses from noise-exposed (red) and control animals (black). The two bottom rows show the performance difference between control and noise-exposed, with green signifying better discrimination performance for noise-exposed, and blue better discrimination performance for control.
